# Treating allergic bronchopulmonary aspergillosis with hormone therapy: natural disease course, regimens, compliance, and outcomes

**DOI:** 10.3389/fmed.2026.1760447

**Published:** 2026-05-13

**Authors:** Qiyin Wu, Yaping Huang, Jiachao Qi, Miaofen Hu, Huixue Zeng, Weiliang Zhang, Liangji Zhang

**Affiliations:** Department of Respiratory and Critical Care Medicine, Zhangzhou Hospital, Fujian Medical University, Zhangzhou, China

**Keywords:** aspergillus, eosinophil, exacerbation, IgE, natural course

## Abstract

**Objective:**

Current guidelines lack information on the natural course and prognosis of allergic bronchopulmonary aspergillosis (ABPA). In this retrospective study, the natural course of ABPA was evaluated in patients treated with hormone therapy.

**Materials and methods:**

25 ABPA patients were given standardized hormone therapy at stage 1 (acute) and stage 3 (exacerbation) of the natural course of disease. Total 47 sessions were divided into 2 groups according to stages, including 25 for stage 1 and 22 for stage 3 ABPA. Followed up once every 2 weeks during the first 8 weeks of treatment. Changes in eosinophils (Δeosinophils) and total serum immunoglobulin E (ΔIgE) were recorded. The correlation between ΔIgE and Δeosinophils at 2 and 4 weeks after treatment was calculated using Kendall’s tau-b (K) correlation analysis.

**Results:**

Some patients did not receive standardized hormone therapy after 4 weeks. After 6 weeks, only 31.91% sessions adhered to standardized hormone therapy. Serum IgE differed between stage 1 and stage 3 patients under the same hormone therapy. Overall, 90.90% of the first ABPA exacerbations occurred within 2 years of diagnosis. The correlation coefficients between ΔIgE and Δeosinophils during treatment were 0.193 and 0.074 at 2 and 4 weeks of treatment, respectively.

**Conclusion:**

The compliance of patients with ABPA to hormone therapy is poor after 4 weeks of treatment. Repeated exacerbations can occur within 2 years of hormone therapy, and ΔIgE should be noted during this period. More evidence is needed to determine whether hormone therapy regimens should be distinguished between stage 1 and stage 3 ABPA.

## Introduction

Allergic bronchopulmonary aspergillosis (ABPA) is an allergic disease caused by hypersensitivity to *Aspergillus fumigatus* (*A. fumigatus*). Owing to the lack of a gold standard for ABPA diagnosis, the rate of underdiagnosis is high, and only a few cases of ABPA have been described in the literature (1). After a diagnosis of ABPA is confirmed, treatment options include hormones, antifungal drugs, and biologics, which are mostly based on expert recommendations, consensuses, and guidelines; however, there are no uniform standards. Additionally, there is a lack of evidence-based post-treatment management protocols (1–4).

In 2016, the International Society for Human and Animal Mycology (ISHAM) released a revised version of the 2013 criteria for ABPA (referred to as the 2016 revised ISHAM criteria), which defines that the natural course of ABPA can be divided into six stages, of which stage 1 is the acute stage (previously not diagnosed as ABPA) and stage 3 is the exacerbation stage (with clinical symptoms and/or new pulmonary infiltrates) (5, 6). Many patients with stage 1 ABPA have recurrent exacerbations even after initial hormone therapy. As a result, the natural course of ABPA is long and complex, and currently available guidelines lack information on the natural course and prognosis of this condition. Additionally, various indicators, including total serum immunoglobulin (Ig)E, eosinophils, and *A. fumigatus*-specific IgG and IgE, lack uniform standards and relevant data on changes in these biomarkers with the natural course of disease (7–9). Furthermore, the treatment regimen for ABPA did not adapt to changes in disease progression, as the hormone therapies used for stages 1 and 3 were consistent (10, 11).

Understanding the acute attack characteristics of the first stage of the natural course of ABPA is crucial for early diagnosis. Similarly, understanding the worsening changes of the third stage can help reduce repeated deterioration of the disease, alleviate patient pain, and reduce social burden. Based on this background, this study aimed to investigate the clinical characteristics of APBP patients with different natural disease sessions, particularly compliance, time of recurrence and exacerbation, and fluctuation levels of important biomarkers such as IgE and eosinophils in patients with stage 1 and stage 3 ABPA. This study also sought to identify differences and correlations between disease sessions. The findings of this study will provide a basis for adjusting ABPA hormone therapy according to disease progression.

## Methods

### Study design

In this retrospective study, patients with newly diagnosed ABPA who were admitted to Zhangzhou Hospital of Fujian Medical University during the period from May 2019 to September 2023 were enrolled. All patients have signed informed consent forms.25 ABPA patients were given standardized hormone therapy at stage 1 and stage 3 of the natural course of disease. Totally 47 sessions were divided into 2 groups according to stages, including 25 for stage 1 and 22 for stage 3 ABPA. As the 2016 revised ISHAM criteria clearly state that there is no widely recognized dosing regimen for oral glucocorticoids in patients with ABPA, corticosteroids are used according to the guidelines and expert recommendations in local areas, with the most widely used regimens being low-dose regimens (5, 6). In the present study, the 2017 Chinese expert consensus on the diagnosis and treatment of allergic bronchopulmonary aspergillosis (12) was applied, with standardized hormone therapy for stage 1 and stage 3 ABPA being oral prednisone 0.5 mg/kg per day for 2 weeks followed by 0.25 mg/kg per day for 4–6 weeks (6, 13). After this initial reduction, the dose was further decreased by 5–10 mg every 2 weeks according to the clinical condition, followed by alternate-day administration. The follow-up results were analyzed.

### Subjects and disease staging

The diagnosis and staging of ABPA were based on the 2016 revised ISHAM criteria.

The diagnostic criteria are as follows: (i) a diagnosis of bronchial asthma, cystic fibrosis, or other pulmonary diseases; (ii) elevated sIgE (anti-*Aspergillus fumigatus*) > 0.35 kUA/L (those diagnosed clinically, but without these data, could also be included if they met all other criteria); total IgE > 1,000 IU/mL (or <1,000 IU/mL if all other criteria were met); (iii) at least two of the following three additional criteria: IgG antibodies against *A. fumigatus* in the serum (>27 mg/L), thoracic radiographic opacities consistent with ABPA (transient or permanent), or a total eosinophil count >500 cells/μL in oral steroid-naïve patients. The exclusion criteria were (i) not meeting the inclusion criteria mentioned above; (ii) combinations of other allergic diseases, except respiratory diseases (6).

The natural course of ABPA can be divided into seven stages: stage 0: asymptomatic and no previous diagnosis of ABPA; stage 1: acute and no previous diagnosis of ABPA (can be further divided into 1a and 1b according to the presence or absence of sputum impaction); stage 2: response (clinical and/or radiological improvement and a decline in IgE by ≥25% from baseline at 8 weeks); stage 3: exacerbation (worsening of clinical symptoms and/or pulmonary manifestations and a ≥50% increase in total serum IgE); stage 4: remission (clinical and radiological improvement after discontinuation of corticosteroids, with total serum IgE fluctuating by <50% at the new baseline level for more than 6 months); stage 5a: treatment-dependent ABPA (>2 recurrences within 6 months after stopping treatment, or clinical, radiological, and/or immunological exacerbations during the administration of oral hormones or azole antifungals); stage 5b: glucocorticoid-dependent asthma (although active ABPA [lung radiological manifestations and serum IgE] is controlled, oral corticosteroids are still required to control refractory asthma); stage 6: advanced ABPA (complicated by type II respiratory failure and/or cor pulmonale, along with radiological findings of pulmonary fibrosis) (5, 6, 8, 12).

### Data acquisition

Serum total IgE was detected by Phadia 100 automatic *in vitro* immunodiagnostic instrument (Phadia AB, Sweden). Automatic modular blood analyzer XN-10[B4] (sysmex corporation) was used to determine peripheral blood eosinophils. The data were mainly obtained from the electronic medical records at our hospital and from the laboratory test sheets of local hospitals during the follow-up period. The data included (i) post-treatment compliance, including the rate of standard guideline-recommended medications and the rate of follow-up testing; and (ii) change in serum total IgE (ΔIgE) and change in blood eosinophils (Δeosinophils) before and after standardized treatment. According to the 2016 revised ISHAM criteria, IgE 417 IU/mL and eosinophils 500 cells/μL were used as the scoring criteria for ABPA, and eosinophils 500 cells/μL was also used as an auxiliary diagnostic cut-off value for ABPA (10). Accordingly, ΔIgE and Δeosinophils were divided into different levels. There were five levels of ΔIgE: level 1: <−417 IU/mL; level 2: −417 IU/mL (included) to 0 IU/mL (not included); level 3: 0 IU/mL; level 4: 0 IU/mL (not included) to 417 IU/mL (included); and level 5: >417 IU/mL. There were five levels of Δeosinophils: level 1: <−500 cells/μL; level 2: −500 cells/μL (included) to 0 cells/L (not included); level 3: 0 cells/L; level 4: 0 cells/L (not included) to 500 cells/μL (included); level 5: >500 cells/μL. For the absolute values of serum total IgE at week 0, week 2 and week 4, four grades were defined: grade 1 (<417 IU/mL), grade 2 (417–1,000 IU/mL), grade 3 (1,000–3,000 IU/mL), grade 4 (>3,000 IU/mL). The absolute values of peripheral blood eosinophil count at the above time points were classified into three grades: grade 1 (<500 cells/L), grade 2 (500–1,000 cells/L), grade 3 (>1,000 cells/L).

### Statistical analysis

Continuous variables are expressed as the mean ± standard deviation or median (interquartile range [IQR]), and categorical data are expressed as number (percentage). Kendall’s tau-b (K) correlation analysis was used to assess statistical associations based on the ranks of the data. *p* < 0.05 (two-tailed) was considered statistically significant. All statistic analyses were performed using IBM SPSS 22.0.

### Ethics approval

This study was performed in accordance with the principles of the Declaration of Helsinki and was approved by the Institutional Ethics Committee of Zhangzhou Hospital Affiliated to Fujian Medical University (No. 20200128).

## Results

### Clinical characteristics and compliance after standardized hormone therapy

A total of 47 sessions of hormone therapy were performed in 25 patients, including 25 sessions of phase 1 (acute) and 22 sessions of phase 3 (exacerbation). The mean age of the study population was 63.88 years; 19 were men (Table 1). In the first 4 weeks of treatment, all patients received standardized hormone therapy in 47 sessions and underwent follow-up laboratory testing once every 2 weeks. After 6 weeks, however, only 15 sessions (31.91%) adhered to the standardized hormone therapy, while 6 sessions (12.77%) continued to cooperate with the follow-up blood tests. After 8 weeks, compliance further decreased, and only 8 sessions (17.02%) continued to adhere to the standardized hormone therapy. The rate of compliance was even lower for patients with stage 3 ABPA than for those with stage 1 ABPA: all patients with stage 3 ABPA refused to undergo follow-up tests after 6 weeks of treatment (Table 2).

**Table 1:** Baseline characteristics of the study population (*n* = 25).

Clinical features	Value
Age, years	63.88 (57.74–70.02)
Male sex	19 (76.00%)
Patient with exacerbation	11 (44.00%)
Patient with multiple exacerbations	5 (20.00%)

All values are presented as numbers (percentages) or mean (95% confidence intervals).

**Table 2:** Proportion of patients who received standardized therapy and who underwent regular follow-up laboratory tests (*n* = 47 sessions of hormone therapy).

	Standardized hormone therapy	Regular follow-up laboratory tests
Overall (stage 1 + stage 3)	*n* (%)	*n* (%)
Initiation	47 (100.00%)	47 (100.00%)
After 2 weeks	47 (100.00%)	47 (100.00%)
After 4 weeks	47 (100.00%)	47 (100.00%)
After 6 weeks	15 (31.91%)	6 (12.77%)
After 8 weeks	8 (17.02%)	6 (12.77%)
Stage 1	*n* (%)	*n* (%)
Initiation	25 (100.00%)	25 (100.00%)
After 2 weeks	25 (100.00%)	25 (100.00%)
After 4 weeks	25 (100.00%)	25 (100.00%)
After 6 weeks	10 (40.00%)	6 (24.00%)
After 8 weeks	5 (20.00%)	6 (24.00%)
Stage 3	*n* (%)	*n* (%)
Initiation	22 (100.00%)	22 (100.00%)
After 2 weeks	22 (100.00%)	22 (100.00%)
After 4 weeks	22 (100.00%)	22 (100.00%)
After 6 weeks	5 (22.73%)	0 (0.00%)
After 8 weeks	3 (13.64%)	0 (0.00%)

Data are shown as *n* (%).

### Overall post-treatment changes in IgE and eosinophils

During the 47 sessions of hormone therapy, 87.23% of the patients refused to undergo follow-up laboratory testing after 4 weeks of treatment; therefore, only the results of the follow-up tests in the first 4 weeks were complete. The serum IgE levels further declined after 2 and 4 weeks of treatment. More than 25% reduction in total serum IgE levels (ΔIgE ≥ 25%) was observed in 38.30% of patients at 2 weeks and 68.09% at 4 weeks following treatment. At 4 weeks of treatment, more than half of the patients had achieved total serum IgE levels in stage 2 (i.e., clinical and serologic remission). Eosinophils also showed a gradual downward trend at treatment initiation. After 4 weeks of treatment, however, the Δeosinophils was quite close to 0 (−0.01), with a positive three-quarter-point value (0.06), indicating that eosinophils began to gradually increase after 4 weeks of treatment (Table 3).

**Table 3:** Overall post-treatment changes in IgE and eosinophils.

Item	IgE (IU/mL)	Eosinophils (10^9^/L)
Initiation	3,420 (2,575, 4,740)	0.62 (0.21, 0.93)
After 2 weeks of treatment	2,840 (1,785, 3,835)	0.35 (0.10, 0.65)
Change in 2 weeks (ΔIgE/Δeosinophils)	−640 (−1,015, −310)	−0.08 (−0.29, −0.02)
ΔIgE ≥ 25% (2 weeks)	18 (38.30%)	–
After 4 weeks of treatment	2,290 (1,265, 3,015)	0.29 (0.11, 0.52)
Change in 4 weeks (ΔIgE/Δeosinophils)	−440 (−815, −80)	−0.01 (−0.07, 0.06)
ΔIgE ≥ 25% (4 weeks)	32 (68.09%)	–

Data are shown as median (interquartile range) or *n* (%). Eosinophils 0.5 = 500 cells/μL.

### Differences in IgE and eosinophils between stage 1 and stage 3 under the same hormone therapy

**Figure 1:**
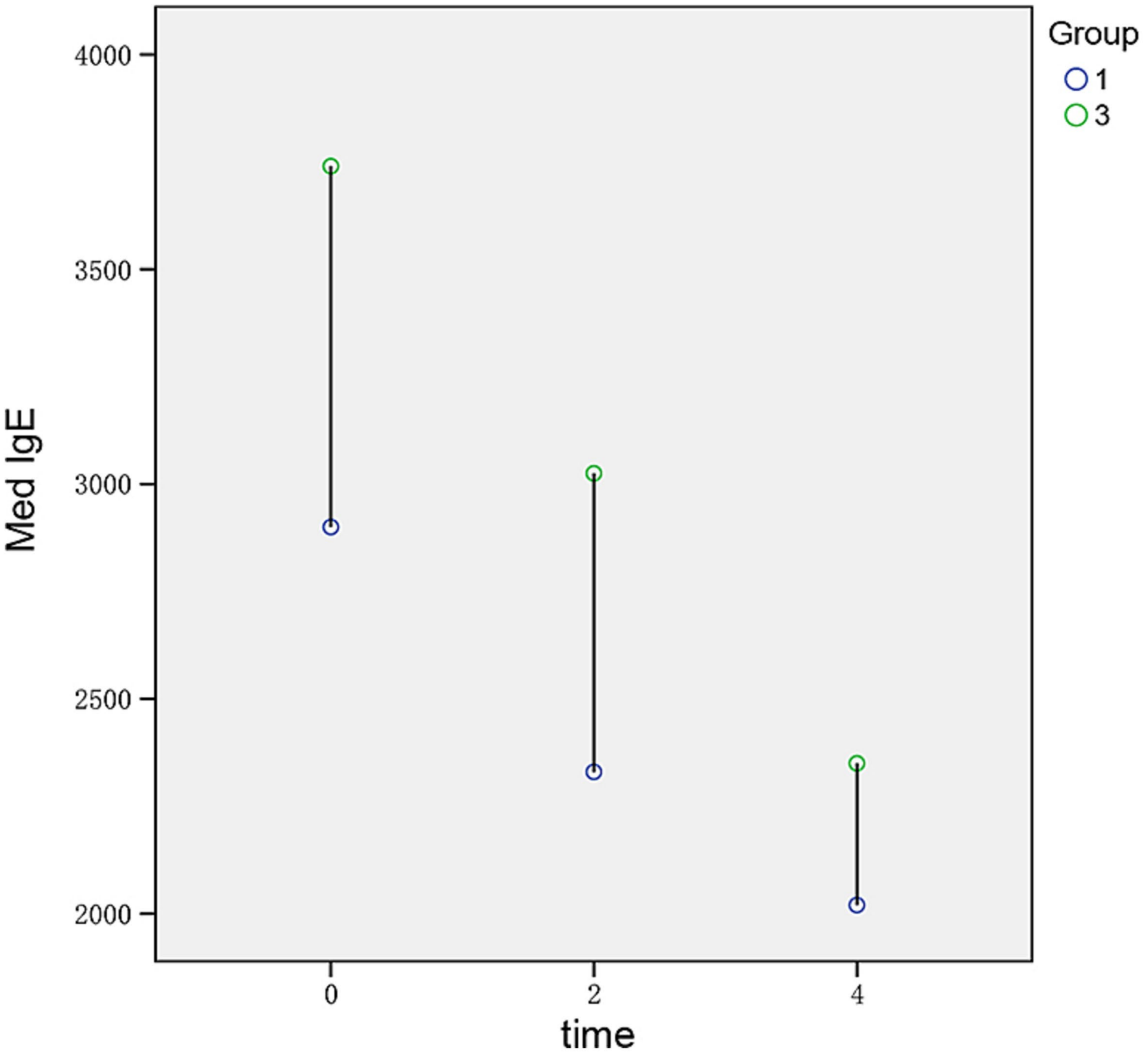
Differences in IgE between stage 1 and stage 3 ABPA under the same hormone therapy at different time points.

**Figure 2:**
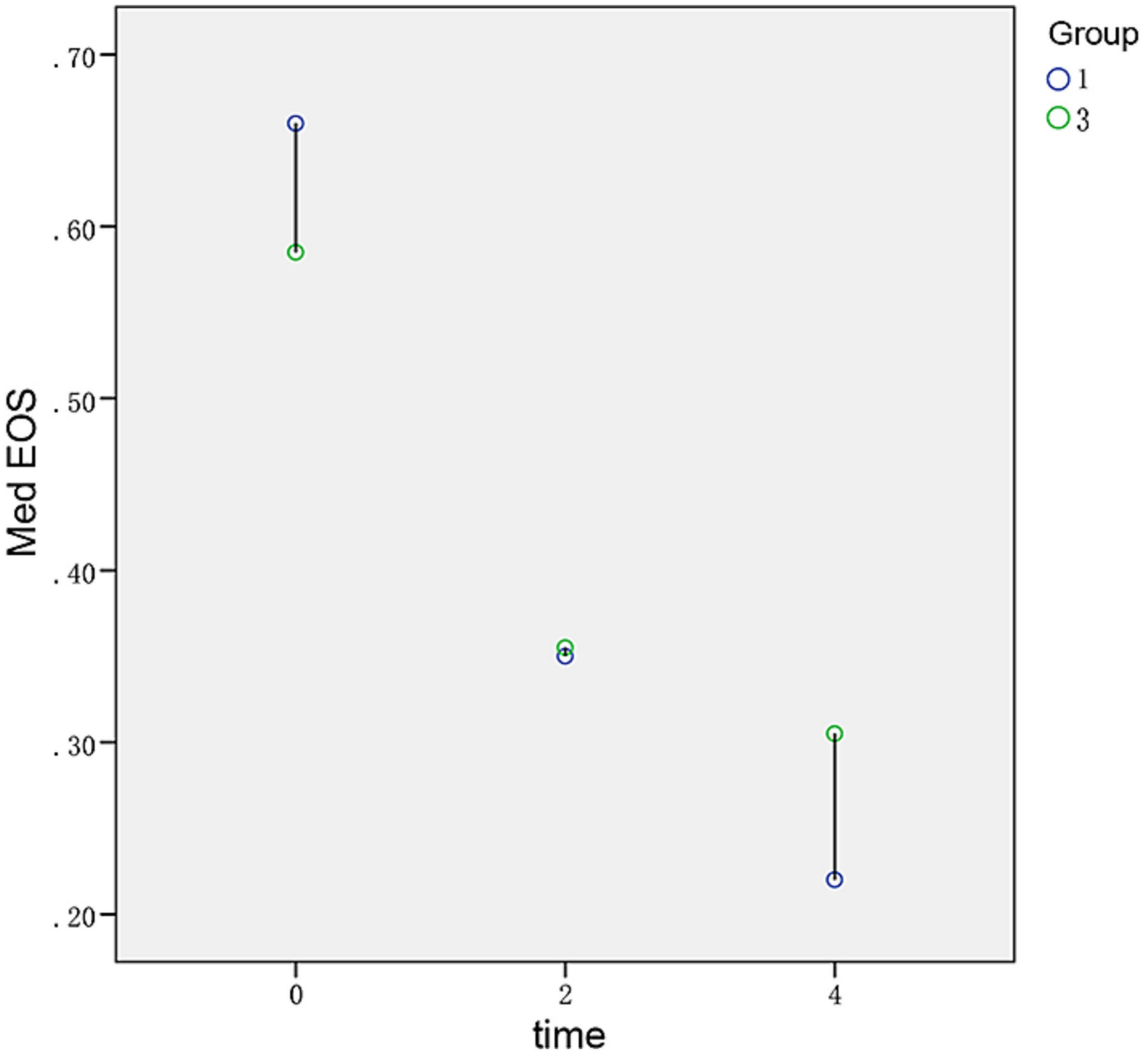
Differences in the levels of eosinophils between stage 1 and stage 3 under the same hormone therapy at different time points.

The median IgE values in patients with stage 1 ABPA were 2,900 (IQR 2,260, 4,770), 2,330 (IQR 1,450, 3,960), and 1915 (IQR 1,132.50, 2,907.50), at initiation, 2 weeks after treatment, and 4 weeks after treatment, and the median IgE values in patients with stage 3 ABPA were 3,740 (IQR 2,895, 4,590), 3,025 (IQR 2,525, 3,687.50), and 2,350 (IQR 1,552.50, 3,032.50), respectively. The serum IgE level was lower in patients with stage 1 ABPA than in those with stage 3 ABPA, but there was no significant difference in eosinophils: Especially 2 weeks after treatment, the median eosinophils values in patients with stage 1 was 0.35 (IQR 0.12, 0.60), and the median eosinophils values in patients with stage 3 eosinophils was 0.30 (IQR 0.06, 0.60). However, statistical analysis of the difference in serum IgE between stage 1 and stage 3 was not possible owing to the different sample sizes between these two stages and the small overall sample size of the study (Figures 1, 2).

### Exacerbations during treatment

Eleven of 25 patients experienced exacerbations, among whom 5 patients had recurrent exacerbations (Table 1). The median time to exacerbation was 13 months, and 90.90% of the first ABPA exacerbations (the first exacerbation after treatment for the first acute onset) occurred within 2 years after the first diagnosis.

### Correlation between ΔIgE and Δeosinophils

IgE and eosinophils changed dynamically during treatment. Kendall’s tau-b correlation was used to analyze the relationship between ΔIgE and Δeosinophils at 2 and 4 weeks. No significant correlation was observed between ΔIgE and Δeosinophils at either time point (*τb* = 0.193 and 0.074, both *p* > 0.05) (Table 4).

**Table 4:** The correlation between ΔIgE and Δeosinophils.

Kendall’stau-b (K)	Time	Correlation coefficient	Significance (double tail)
	2 weeks	0.193	0.157
	4 weeks	0.074	0.561

Intra-patient temporal correlations of IgE and eosinophils were analyzed at week 0, week 2 and week 4. Significant positive correlations were observed for IgE between week 0 and 2 (*τb* = 0.840, *p* < 0.05) and between week 2 and 4 (*τb* = 0.586, *p* < 0.05). For eosinophils, significant positive correlations were also found between week 0 and 2 (*τb* = 0.641, *p* < 0.05) and between week 2 and 4 (*τb* = 0.790, *p* < 0.05). These results indicated consistent longitudinal changes of both markers during initial Stage-I treatment.

## Discussion

In this study, long-term adherence to corticosteroid therapy for ABPA was poor, and maintaining guideline-based treatment was difficult in clinical practice (9). This may be related to patient self-discontinuation due to symptom improvement, concerns about corticosteroid side effects, and insufficient awareness of maintenance treatment and maintaining guideline-based treatment was difficult in clinical practice. Patients showed the best compliance during the first 4 weeks of treatment, making this period critical for therapeutic success. Despite repeated education on standardized medication and regular follow-up in non-primary hospitals, most patients failed to sustain adherence after 4 weeks. Therefore, more effective strategies are needed to strengthen follow-up during the initial 4 weeks and extend this high-compliance period. It can be reasonably assumed that patient in primary hospitals adherence might have declined further in the real-world clinical. The Chinese expert consensus on the diagnosis and treatment of allergic bronchopulmonary aspergillosis (2022 update) clearly states that there is a lack of standardized treatment regimens for ABPA. Despite research advances in ABPA, such as the underlying mechanism of heterozygous *CARD9* mutations (14); the prophylactic effect of amphotericin (15); and the application of biologics, such as omalizumab and mepolizumab (3, 16), all available antifungal drugs and biologic treatments are still based on hormone therapy, highlighting the importance of learning the long-term outcomes of hormone treatment in patients with ABPA. As there is a lack of a globally recognized hormone therapy program for ABPA, it is important to develop appropriate and feasible hormone use protocols. In the present study, 68.09% of patients with ABPA had a decrease of ≥25% in serum IgE after 4 weeks of standardized hormone therapy (in accordance with the 2017 expert consensus), reaching stage 2 (i.e., clinical and serologic remission). Unfortunately, most patients paid less attention to ABPA and refused to receive standardized drug therapy and to undergo laboratory testing after their symptoms improved, which was a typical manifestation of the decline in compliance after 4 weeks of hormone treatment. Therefore, an applicable hormone therapy protocol should also focus on treatment adherence after 4 weeks of treatment in addition to the first 4 weeks, so as to improve the efficacy of standardized hormone therapy for patients with ABPA. However, the current study was limited by its single-center design, and multicenter studies are needed to obtain more evidence.

The hormone treatment protocols for stage 1 and stage 3 ABPA are the same in the 2017 Chinese expert consensus and in the 2016 revised ISHAM criteria (6, 12). However, the present study revealed that patients with stage 1 or stage 3 ABPA had different adherence to the same treatment regimen, along with different IgE levels. Thus, different hormone treatment regimens may be required depending on the course of the disease. The present study showed that patients who received standardized hormone therapy in stage 1 were less compliant when their disease entered stage 3, which may explain the higher IgE levels in stage 3 among these patients. IgE-mediated type I hypersensitivity plays a central role in the pathogenesis of ABPA (17), and the change in IgE may reflect the evolution of the disease. In the present study, the median total serum IgE of patients in stage 3 was higher than that of patients in stage 1, both before and after treatment, although it was not possible to statistically compare the difference in serum IgE between stage 3 and stage 1 owing to the difference in the sample size. Studies with larger sample sizes are needed to further explore the potential difference in the level of IgE between stage 1 and stage 3, and more studies are warranted to investigate whether the use of hormones during stage 1 leads to drug resistance during stage 3.

ABPA is a long-term chronic disease that does not evolve in a sequential manner. Repeated exacerbations can occur even after standardized hormone therapy. Notably, the vast majority of first ABPA exacerbations occur within the first 2 years after treatment initiation. Similarly, Agarwal et al. (7) proposed that ABPA exacerbations were common within 3 years after treatment cessation. However, the present study suggested that the first ABPA exacerbations were mostly seen within 2 years after treatment initiation, which is quite different from the 3-year interval. The reason for such a difference may be that the patients in the present study did not complete hormone therapy in full accordance with the guidelines, whereas patients in other studies took a sufficient amount of hormone therapy. For example, in the study conducted by Agarwal et al. (7), all subjects received oral prednisolone for 4 months as standard. Of course, ABPA exacerbations are complex and can be affected by a variety of factors, such as drug combinations. For instance, itraconazole combined with prednisone for ABPA resulted in a lower rate of acute exacerbations within 1 year of treatment compared with that of prednisone monotherapy, and acute exacerbations were reduced by more than 10% within 1 year (18). In addition, combining Omalizumab resulted in a reduction in oral hormone dosage, fewer acute exacerbations and hospitalizations, and improved lung function in patients with ABPA (19, 20). Other influential factors include the presence of serologic ABPA or ABPA with central bronchiectasis, which respond differently to hormone treatment (21) and age (22). Multicenter studies with large sample sizes are warranted to further analyze the survival of patients with ABPA with exacerbations.

Dynamic changes in IgE may reflect changes in ABPA status. However, eosinophils have low sensitivity and specificity for diagnosing ABPA (13). In addition, the degree of peripheral blood eosinophil infiltration does not parallel that of pulmonary eosinophils. Although some authors have claimed that eosinophils have notable significance for dynamic follow-up (23), the present study showed that the eosinophil count began to increase gradually after 4 weeks of treatment and did not change regularly with treatment during the exacerbation stage. Furthermore, Δeosinophils had a weak correlation with ΔIgE. Therefore, the assessment of eosinophils was not justified to be an effective indicator of exacerbations or for follow-up use.

### Limitations

This study has some limitations that should be considered. First, this was a single-center study with a small sample size, so the findings should be clarified in large-sample studies in the future. Second, there was an inconsistent sample size between phase 1 and phase 3 ABPA, which meant that certain statistical comparisons could not be made.

## Conclusion

The first 4 weeks are the most important period for hormone therapy in patients with ABPA, and the compliance of patients taking hormone therapy should be strengthened after 4 weeks. Hormone therapy regimens may differ between stage 1 and stage 3 ABPA. ABPA exacerbations occur mainly within 2 years of treatment initiation, and monitoring of serum IgE may help to predict the outcomes of the disease.

## Data Availability

The original contributions presented in the study are included in the article/supplementary material, further inquiries can be directed to the corresponding author.
